# Neuropsychiatric Symptoms Exacerbate the Cognitive Impairments in Patients With Late-Life Depression

**DOI:** 10.3389/fpsyt.2021.757003

**Published:** 2021-11-19

**Authors:** Min Zhang, Ben Chen, Xiaomei Zhong, Huarong Zhou, Qiang Wang, Naikeng Mai, Zhangying Wu, Xinru Chen, Qi Peng, Si Zhang, Minfeng Yang, Gaohong Lin, Yuping Ning

**Affiliations:** ^1^The Affiliated Brain Hospital of Guangzhou Medical University, Guangzhou, China; ^2^The First School of Clinical Medicine, Southern Medical University, Guangzhou, Guangdong, China; ^3^Guangdong Engineering Technology Research Center for Translational Medicine of Mental Disorders, Guangzhou, China

**Keywords:** late life depression, cognitive function, neuropsychiatric symptoms, mediating effect, moderating effect

## Abstract

**Background:** Neuropsychiatric symptoms (NPS) and cognitive impairments are both common in patients with late-life depression (LLD). However, the relationship between NPS and cognitive functions in LLD patients remains unclear. The current study aims to explore the effects of NPS on cognitive impairments in LLD patients.

**Methods:** Two hundred and sixty-two LLD patients and 141 normal controls (NC) were recruited. Exploratory factor analysis was used to extract factors from the Neuropsychiatric Inventory (NPI). Correlation, mediation, and moderation analyses were used to explore whether NPS exacerbated the cognitive impairments in LLD and whether NPS exhibited different effects on cognitive impairments in acute-state LLD (aLLD) and recovery-state LLD (rLLD).

**Results:** Three main factors were extracted from the NPI, including emotional, behavioral, and psychotic factors. The patients with LLD exhibited worse cognition and higher NPI scores, and the scores of NPI-total and three extracted factors were negatively associated with cognitive scores. The mediation analyses exhibited that NPI-total and behavioral factor scores increase the difference in cognition scores between LLD and NC groups. The mediation analyses exhibited that behavioral factor score played a greater effect on impairing MMSE in the rLLD group than in the aLLD group. Additionally, behavioral factor score was in a trend to be negatively associated with Mini-Mental State Examination (MMSE) score changes at a one-year follow-up (*p* = 0.051).

**Conclusions:** NPS, especially behavioral symptoms, exacerbate cognitive impairments in LLD and may contribute to residual cognitive impairment in rLLD patients. Early intervention for behavioral symptoms in LLD patients may be beneficial to their long-term clinical prognosis.

## Introduction

Late-life depression (LLD), defined as major depression occurring in an older adult (60 years or older), is a global public health problem that severely limits psychosocial function and increases mortality (1, 2). In addition to persistent low mood and decreased activation, cognitive impairments (such as impairments in information processing speed, episodic memory, and executive function) are also common in LLD patients (3–5). Additionally, sustained cognitive deficits in individuals suffering from LLD have been associated with higher depression relapse rates, poorer responses to antidepressant treatment, accelerated rates of functional decline, and progression to dementia (5–9). A previous study suggested that cognitive impairments persisted in 94% of LLD patients with cognitive deficits at baseline, despite have reached depression remission (10), suggesting that there may be other factors influencing cognition other than depression. Therefore, it is important to explore which other factors may influence cognitive function in patients with LLD because they may inform early intervention and lead to better prognosis.

Neuropsychiatric symptoms (NPS) are non-cognitive, behavioral, or psychiatric symptoms such as aberrant motor behavior, irritability, anxiety, and hallucinations (11). NPS are associated with a higher burden of neuropathologic markers of dementia (12), more brain lesions (13), worse cognitive functions (14), greater functional impairment (15), and poorer quality of life (16). Large-sample longitudinal studies also supported the idea that NPS such as anxiety, apathy, and nighttime behaviors were associated with a more rapid rate of cognitive decline (17–20). Specifically, Palmer et al. found that apathy, but not depression, was associated with progression from amnestic-MCI to dementia, indicating that there may be a differential impact of different NPS on the dementia course (21). Patients with LLD often undergo aging of the brain with deterioration of the cerebral white matter, measured by white matter hyperintensities (WMH). WMH were found to be associated with significant preclinical NPS as well as hippocampal atrophy (22), which is considered a risk factor for developing cognitive impairment. Moreover, in a recent systematic review, Piras et al. identified several potential factors with confounding effects on the risk association between depression and dementia, including hypothalamic-pituitary-adrenal axis dysfunction, activation of inflammatory pathways, neuroanatomic changes, vascular risk, and metabolic and genetic factors (23). Thus, although the relationship between cognition and NPS is not entirely clear, numerous investigations have shed some light on the potential neurobiological links between NPS and cognitive impairments, as discussed above. In short, previous studies have pointed out that NPS were strongly correlated with cognitive decline across the spectrum from normal cognition to dementia (24), implying that NPS might be one of the risk factors for dementia.

Unlike the form of depression that occurs in young adults, LLD patients show more somatic symptoms and NPS (25, 26). Moreover, LLD patients with NPS always exhibit poorer clinical prognoses. For instance, apathy and reduced appetite in LLD were found to be associated with an increased risk of all-cause mortality (27). Comorbid anxiety in LLD has been found to increase the burden of depression, as reflected by quality of life, physical disability, and increased health care use (28). Ji et al. suggested that all insomnia symptoms were positively associated with depressive symptoms and that nighttime insomnia symptoms were indicative of poor cognitive performance (29). Bingham et al. found an association between cerebrovascular risk and treatment outcomes of LLD patients with psychotic features (30).

However, a limited number of studies to date have comprehensively evaluated the various kinds of NPS in patients with LLD, and the relationships between NPS and cognitive function in LLD patients has not been fully elucidated. Thus, the present study aimed to explore the relationships between NPS and cognitive impairment in LLD patients by using mediation and moderation analyses. Based on what has been mentioned above, it was hypothesized that NPS exacerbate cognitive impairments in LLD patients and contribute to persistent cognitive impairment during recovery periods. This study provides a deeper understanding of how NPS and cognitive impairment interact with each other and provides new insights into the treatment strategies of cognitive impairment in LLD patients.

## Materials and Methods

### Participants

Two hundred sixty-two LLD patients were continuously recruited from The Affiliated Brain Hospital of Guangzhou Medical University (Guangzhou Huiai Hospital), and 141 normal controls (NCs) were recruited from the communities in Guangzhou, China. All participants or their legal guardians provided written informed consent to take part in the study. This study was approved by the ethics committees of The Affiliated Brain Hospital of Guangzhou Medical University.

The inclusion criteria for patients with LLD were as follows: the patients were ≥60 years of age, had at least one episode of depression after the age of 60, and met the criteria for major depression in the *Diagnostic and Statistical Manual of Mental Disorders, Fourth Edition*. The NC subjects were aged 60 years or older without psychiatric disorders and exhibited normal cognitive function. The cut-off scores to classify cognition as normal for the NC subjects were based on MMSE thresholds adjusted by their years of education. For subjects who were illiterate, the cut-off was 17 points; those who received education through primary school had a cut-off of 20 points; and those who received education equal to and over middle school had a cut-off of 24 points. In addition to the MMSE, NC subjects were also evaluated by other scales, such as ADL, to exclude those with possible cognitive impairment.

All participants were evaluated by at least two psychiatrists to assess their clinical characteristics. The exclusion criteria of all participants were as follows: (1) history of other major psychiatric disorders, such as bipolar disorder and schizophrenia; (2) family history of schizophrenia and bipolar disorders; (3) physical illness that may induce emotional abnormalities, such as anemia and hypothyroidism; (4) neurological disease, such as brain tumor and stroke; and (5) drug or alcohol use disorders.

The severity of depressive symptoms was measured using the 17-item Hamilton Depression Rating Scale (HAMD-17). The patients with LLD were further divided into two groups based on HAMD scores: recovery-state LLD (rLLD) with HAMD <17 and acute-state LLD (aLLD) with HAMD ≥17 (31).

### Neuropsychological Assessments

The participants underwent a full-scale battery of neuropsychological tests to evaluate cognitive functions. The battery included the following: (1) global cognition: Mini-Mental State Examination (MMSE); (2) memory: auditory verbal learning test (AVLT) and Rey-Osterrieth Complex Figure (ROCF)-delayed recall test; (3) language ability: Boston naming test (BNT) and verbal fluency test (VFT); (4) information processing speed: symbol digit modalities test (SDMT) and Trail Making Test (TMT)-A; (5) executive function: Stroop color and word test (SCWT)-C and TMT-B; and (6) visuospatial skill: ROCF-copy test and clock drawing test (CDT). The scores in each cognitive domain were calculated by transforming each of the test scores to standardized z scores and summing the scores from the two tests. Notably, for the tests measured by timing, including the SCWT-C, TMT-A, and TMT-B, lower scores indicated better performance. Thus, the scores were converted to the reciprocal before they were converted to the standard score (32).

### Neuropsychiatric Assessment

Neuropsychiatric symptoms were assessed by using the Neuropsychiatric Inventory (NPI), which includes delusions, hallucinations, agitation, depression, anxiety, euphoria, apathy, disinhibition, irritability, aberrant motor behavior (Ab. Mot Beh), sleep disturbances, and eating disturbances (33). The presence of symptoms for each item during the past 30 days was asked with a screening question. Once this was endorsed, specific questions were asked to clarify their frequency, severity, and burden for caregivers. Each item's score could range from 0 to 12 and reflected both severity and frequency ratings, with 0 corresponding to the absence of symptoms and 12 corresponding to its maximum frequency and severity.

### Statistical Analyses

Statistical Package for Social Sciences version 23.0 (IBM SPSS 23.0, Chicago, IL, USA) was used to perform the statistical analyses. Demographic and clinical variables were analyzed using *t*-tests and one-way analysis of variance (ANOVA) for continuous variables, and chi-square (χ^2^) tests were used for categorical variables. Cognitive functions were compared with analysis of covariance (ANCOVA). Control variables included age, sex, and education years. Statistical significance was defined as *p* <0.05.

Preliminarily, 12 *t*-tests were used to assess between-group differences in NPI. Exploratory factor analysis was subsequently used to extract the main factors from the NPI for all of the participants. Factor analysis describes variability among observed correlated variables in terms of a potentially lower number of unobserved variables called factors. In the present study, we hypothesized that the factors represented the common variance in the 12 items of the NPI. Each item score was entered as a variable of interest in the factor analysis. Factor analysis was performed using the principal component estimation method (with eigenvalues >1) and VARIMAX method for factor rotation. Factors extracted from the NPI were then used in partial correlation, regression, mediation, and moderation analyses. Partial correlation analyses were used to explore the correlations between NPS and cognitive functions after adjusting for age, sex, and education years, and the regression analyses were used to further explore the significance of the correlations. As for the follow-up data, partial correlation analyses were used to explore the correlations between NPS and MMSE scores change after adjusting for age, sex, and education years.

Mediation analyses were performed for NPI-total scores and main factors screened in exploratory factor analysis. The mediation model was established when the following conditions were met: (1) the independent variable (IV) had a significant effect on the dependent variable (DV); (2) the IV significantly predicted the mediator; (3) the mediator significantly affected the DV; and (4) exclusion of the mediator from the model decreased the effect of the IV on the DV. For the present mediation analyses, dichotomous variables of NC and LLD groups were regarded as the IV, neuropsychological indicators were regarded as DVs, and NPI-total scores and scores of extracted main factors were regarded as mediators. In addition, for the moderation analyses, dichotomous variables of aLLD and rLLD groups were regarded as the IV, neuropsychological indicators were regarded as DVs, and NPI-total scores and extracted main factors were regarded as moderators. PROCESS 3.2 was used to investigate the mediating and moderating relationships among the variables (34). Indirect effects were estimated with 5,000 bootstrapped samples. Moreover, the Sobel test was performed to verify whether the mediating effect was significant.

## Results

### Demographic and Clinical Characteristics, Neuropsychological Assessment, and Neuropsychiatric Symptoms

Information on the demographics, clinical symptoms, and cognitive functions of all participants is listed in Table 1. Significant differences were found in years of education, HAMD scores, NPI-total scores, MMSE scores, and scores in all domains of cognitive function between the NC group and LLD group (*p* < 0.001). There were significant differences in all of the NPI items (except for disinhibition) between the NC group and LLD group (*p* < 0.05) (Figure 1). Euphoria item has zero variance, and thus is not included in the analysis.

**Table 1:** Demographic, clinical symptoms, and neuropsychological information.

**Variables**	**NC (*n* = 141)**	**LLD (*n* = 262)**	**F/χ^2^/t**	***p* value**
Age (years)	67.72 ± 5.42	68.43 ± 7.04	−1.128	0.260
Sex (male/%)	41 (29.1%)	61 (23.3%)	1.629	0.202
Education years	10.76 ± 3.03	8.11 ± 4.07	7.369	*p* < 0.001
Age of onset (years)	NA	59.94 ± 11.21	/	/
Disease duration (years)	NA	5.41 ± 7.95	/	/
Numbers of episode	NA	2.18 ± 2.45	/	/
HAMD	1.81 ± 2.37	12.17 ± 8.25	−18.902	*p* < 0.001
NPI-total	2.62 ± 5.00	23.60 ± 17.70	−17.792	*p* < 0.001
MMSE^#^	27.28 ± 1.89	21.38 ± 5.49	99.402	*p* < 0.001
Memory^#^	1.04 ± 1.19	−0.88 ± 1.62	115.938	*p* < 0.001
Language ability^#^	1.12 ± 1.00	−0.99 ± 1.57	154.537	*p* < 0.001
Informationprocessing speed^#^	1.01 ± 1.29	−0.82 ± 1.68	97.356	*p* < 0.001
Executive function^#^	0.83 ± 1.38	−0.63 ± 1.56	68.075	*p* < 0.001
Visuospatial skill^#^	0.97 ± 0.62	−0.81 ± 1.91	83.407	*p* < 0.001

*NC, normal control; LLD, late life depression; NA, Not applicable; HAMD, Hamilton Depression Rating Scale; NPI-total, total scores of Neuropsychiatric Inventory; MMSE, Mini-mental state examination*;

# * adjusted for age, sex, education years*.

**Figure 1:**
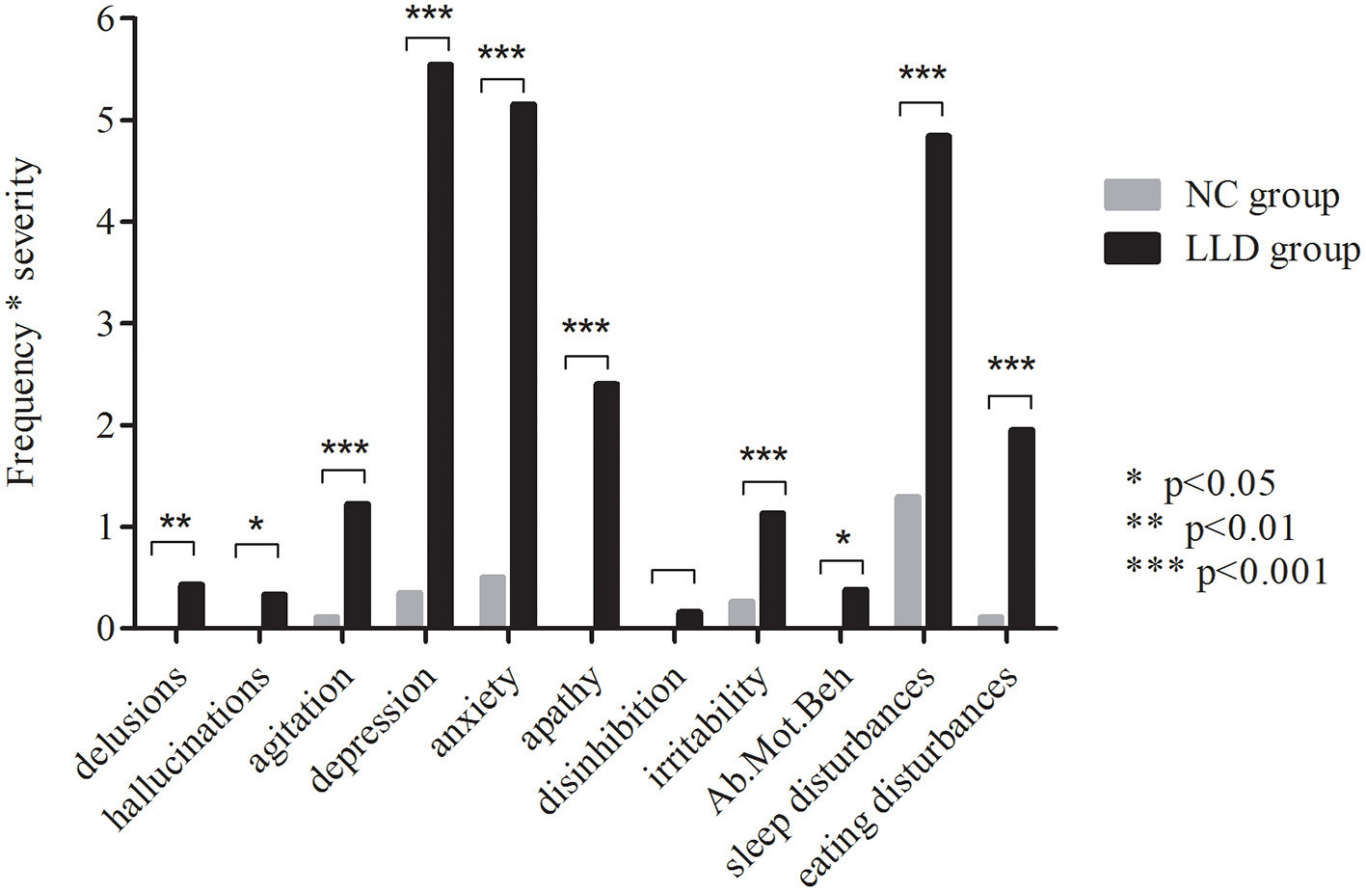
Neuropsychiatric symptoms in NCs and LLD patients. The figure shows the scores of Frequency * Severity in NCs (in gray) and LLD patients (in black), as assessed by NPI; euphoria item has zero variance, thus is not included in the analysis; NC, normal control; LLD, late life depression; Ab. Mot Beh, aberrant motor behavior.

### Factor Analysis

The KMO measure in this study was 0.726, indicating appropriate correlations among NPI items. In addition, Bartlett's test of sphericity showed that statistical significance was less than 0.001, thereby confirming the goodness-of-fit of the model.

Based on the NPS item scores, three factors were extracted by using exploratory factor analysis. The NPI items with a saturation threshold >0.40 were included in 3 factors. Factor 1 described emotional symptoms, which included depression, anxiety, apathy, sleep disturbances, and eating disturbances. Factor 2 described behavioral symptoms, which included agitation, disinhibition, irritability, and Ab. Mot Beh. Factor 3 described psychotic symptoms, which included hallucinations and delusions (Table 2). The variance in Factor 1, Factor 2, and Factor 3 was 2.87, 1.66, and 1.61, respectively. The explanatory power of Factor 1, Factor 2, and Factor 3 was 26.1, 15.1, and 14.6%, respectively. The total explanatory power of the three factors was 55.7% of the total variance.

**Table 2:** Communalities and rotated factor matrix across all subjects.

	**Communalities #**		**Rotated factor matrix**		
	**Initial**	**Extraction**	**Factor 1**	**Factor 2**	**Factor 3**
Delusions	1.00	0.78	0.16	0.20	**0.84**
Hallucination	1.00	0.74	0.11	0.02	**0.86**
Agitation	1.00	0.52	0.23	**0.68**	−0.03
Depression	1.00	0.77	**0.86**	0.14	0.04
Anxiety	1.00	0.65	**0.79**	0.18	−0.01
Apathy	1.00	0.23	**0.45**	0.16	0.06
Disinhibition	1.00	0.58	−0.06	0.72	0.23
Irritability	1.00	0.47	0.36	**0.57**	−0.16
Ab.Mot Beh.	1.00	0.25	0.07	**0.48**	0.11
Sleep disturbances	1.00	0.58	**0.75**	0.03	0.08
Eating disturbances	1.00	0.58	**0.72**	0.03	0.25

*Euphoria item has zero variance, thus not included in the factor analysis; factor 1, emotional factor; factor 2, behavioral factor; factor 3, psychotic factor; Ab. Mot Beh, aberrant motor behavior; # Extraction method: Principle component analysis; Rotation method: Varimax with Kaiser Normalization; in bold the variables included in each factor according the saturation threshold >0.40*.

The three factor scores for the NPI presented for the NC and LLD groups are shown in Supplementary Table 1. Among the three factors, only the emotional factor and behavioral factor scores were significantly higher in the LLD group than in the NC group (*t* = −16.688, *p* < 0.001; *t* = −4.239, *p* = 0.001, respectively).

### Correlations Between NPS and Cognitive Functions

NPI-total scores and emotional factor scores were negatively correlated with MMSE scores and scores in all cognitive function domains. Behavioral factor scores were correlated with MMSE, memory, language ability, and visuospatial skill scores, while psychotic factors were correlated with MMSE and language ability scores (*p* < 0.05) (Table 3). Linear regression showed that the correlations between MMSE, memory, language skill, and NPS scores were maintained, while some of the correlations between other cognitive domains and NPS scores disappeared. The detailed results are shown in Supplementary Table 2.

**Table 3:** Correlations between NPS and cognitive function in all subjects.

	**MMSE**	**Memory**	**Language ability**	**Information processing speed**	**Executive function**	**Visuospatial skill**
NPI-total	−0.397^***^	−0.388^***^	−0.420^***^	−0.382^***^	−0.306^***^	−0.354^***^
Emotional factor	−0.379^***^	−0.355^***^	−0.387^***^	−0.380^***^	−0.308^***^	−0.316^***^
Behavioral factor	−0.184^**^	−0.189^**^	−0.194^**^	−0.087	−0.082	−0.190^**^
Psychotic factor	−0.172^**^	0.112	0.126^*^	0.062	0.087	0.090

*Data are represented with r*,

* *p <0.05*;

** *p <0.01*;

*** *p <0.001, adjusted for age, sex, and education year; NPI-total, total scores of Neuropsychiatric Inventory; MMSE, Mini-mental state examination*.

### Mediation Analysis

**Figure 2:**
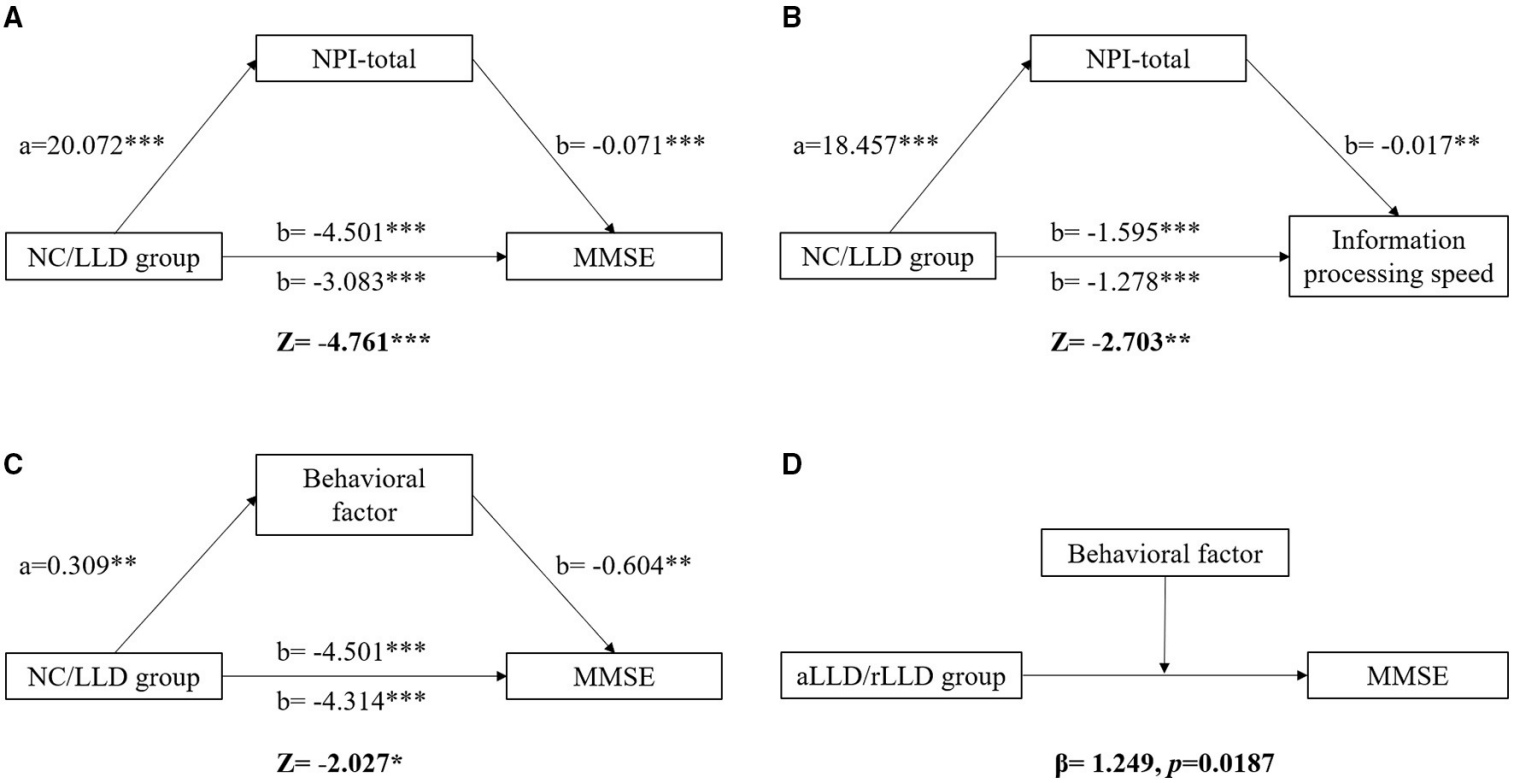
The mediating and moderating effect of NPS on cognition. Differences of MMSE and information processing speed between NC and LLD groups were mediated by NPI-total **(A, B)**. Difference of MMSE between NC and LLD groups was also mediated by behavioral factor **(C)**. The NPI-total and behavioral factor are both the partial mediator for the relationship between NC/LLD groups and the cognitive functions. The MMSE difference between aLLD and rLLD groups was moderated by behavioral factor **(D)**. Behavioral factor played a greater effect on impairing MMSE in rLLD group than in aLLD group. * *p* < 0.05; ** *p* < 0.01; *** *p* < 0.001.

Considering the close relationship between NPS and cognitive functions, the present study subsequently conducted a mediation analysis to explore their relationships. Overall, we discovered three mediation models (Figure 1). First, the total effect of the NC/LLD groups on MMSE scores was β = −4.501 (*t* = −10.012, *p* < 0.001). The indirect effect of groups on MMSE through NPI-total was −1.418 (*z* = −4.761, *p* < 0.001) (Figure 2A). Second, the total effect of the NC/LLD groups on the information processing speed subscale score was β = −1.595 (*t* = −9.654, *p* < 0.001). The indirect effect of groups on information processing speed through NPI-total was −0.317 (*z* = −2.703, *p* < 0.01) (Figure 2B). The above results showed that NPI-total score as a partial mediator exacerbated the cognitive impairments in the LLD group evidenced by reduced MMSE and information processing speed scores.

Since three factors were extracted in the current study, we further explored which factors contributed most to the mediating effect. We found a mediation model involving the behavioral factors. The total effect of the NC/LLD groups on MMSE scores was β = −4.501 (*t* = −10.012, *p* < 0.001). The indirect effect of groups on MMSE through behavioral factor was −0.187 (*z* = −2.027, *p* < 0.05), indicating that the behavioral factor score as a partial mediator exacerbated the cognitive impairments in the LLD group evidenced by reduced MMSE scores (Figure 2C).

No significant mediating effect for the other NPI factors on the association between groups and cognitive functions was found.

### Moderation Analysis

Because of the existence of a mediating effect of NPS on cognition in the LLD patients, the present study subsequently explored the differential effects of NPS on cognition between aLLD and rLLD patients. There were 192 rLLD patients and 70 aLLD patients. The demographics, clinical symptoms, cognitive functions, and NPS for each group are listed in Supplementary Table 3. We discovered only one moderation model. The effects of aLLD/rLLD groups and behavioral factors on MMSE scores were β = −2.258 (*t* = −3.384, *p* < 0.001) and β = −2.524 (*t* = −2.870, *p* = 0.005), respectively, which manifested significant negative effects of both LLD and behavioral factors on MMSE scores. A moderating effect of behavioral factors on MMSE scores between the aLLD and rLLD groups was found, with β = 1.249 (*t* = 2.367, *p* = 0.019), suggesting that the behavioral factor had a greater effect on impairing MMSE scores in the rLLD patients than in aLLD patients (Figure 2D).

Since it is possible that the illness duration has a moderation effect on general cognition, we also performed the moderation analysis of illness duration on MMSE scores between rLLD and aLLD patients. However, we didn't find the moderation effect with β = 0.138 (*t* = 0.775, *p* = 0.439).

### Follow-Up Study of the MMSE

**Figure 3:**
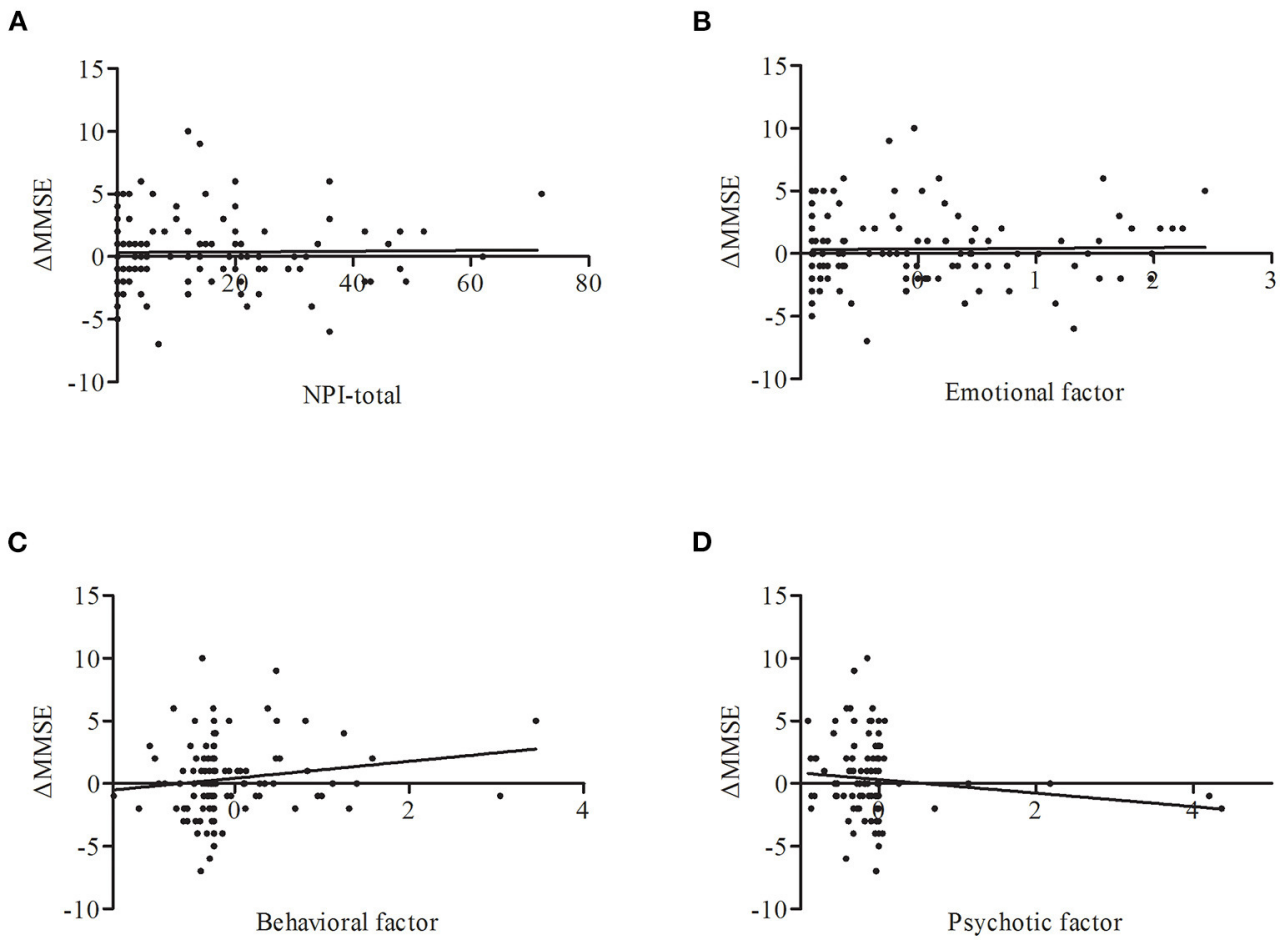
Correlations between ΔMMSE and NPS including NPI-total **(A)**, emotional factor **(B)**, behavioral factor **(C)**, psychotic factor **(D)** across all subjects. Only behavioral factor on baseline has a trend to be correlated with the change of MMSE score (*r* = 0.164, *p* = 0.051). ΔMMSE, MMSE score (baseline)-MMSE score (one year), adjust for age, sex, and education years.

Sixty-one NCs and 85 LLD patients received a follow-up assessment after one year. As the sample size of follow-up is relatively small, we take all of them as a group to perform the next analysis. Partial correlation analysis found that the behavioral factor scores at baseline shown a trend to be correlated with changes in MMSE scores (*r* = 0.164, *p* = 0.051) (Figure 3).

## Discussion

In the present study, we first used the NPI to evaluate NPS in patients with LLD in a large sample size and demonstrated the mediating and moderating effects of NPS on cognitive functions in LLD patients. The main findings were as follows: (1) The LLD group exhibited significantly worse cognitive functions and higher NPS scores than the NC group. (2) NPI-total scores mediated differences in MMSE and information processing speed scores between the NC and LLD group. Moreover, behavioral factor also mediated the difference in MMSE scores. (3) Behavioral factor moderated the difference in MMSE scores between the aLLD and rLLD groups, and was in a trend to be positively associated with changes in MMSE scores at the one-year follow-up.

Previous studies have demonstrated that both LLD and NPS are risk factors for cognitive decline and dementia (17, 35), but the relationship between NPS and cognitive impairment in LLD patients has remained unclear. The present study found that NPS exhibited a negative mediating effect on cognitive function in patients with LLD, suggesting that NPS exacerbated cognitive impairments in LLD patients. It has been repeatedly reported that NPS were associated with the risk of incident MCI or more rapid progression to severe dementia (17, 19, 36). The reasons may be the following: (1) NPS may be a consequence of dementia pathology, such as Alzheimer's disease (AD) (13). The key brain regions underlying the behavioral, emotional, and psychotic factors are affected in patients with AD and LLD, and therefore, NPS might be a very early non-cognitive manifestation of dementia in LLD. (2) NPS may be generated by brain vascular disease and subsequent changes in white matter (e.g., WMH). There is a vascular depression hypothesis of LLD, and vascular lesions in the brain may lead to the occurrence of NPS. (3) Dementia-related biomarkers may be involved. A recent study found that tau protein was deposited in specific brain regions of LLD patients and that the deposition was severe in LLD patients with NPS (34). Tau deposition might etiologically link LLD and NPS. As tau pathogenesis is widely recognized in dementia, it is not surprising that LLD patients with NPS exhibit worse cognition. The present study also found a trend in correlation between behavioral factor scores and changes in MMSE scores at one year, indicating that behavioral symptoms at baseline might contribute to the prediction of future cognitive decline, no matter in LLD patients or NCs. However, these results should be carefully interpreted due to the small sample size and the heterogeneity in the LLD group. Large sample sizes in follow-up studies are needed to explore the predictive power of NPS on global cognitive decline in those with LLD.

The present study extracted three factors from the NPI, which was consistent with some previous studies in patients with AD (37, 38). However, only 55.7% of the variance could be explained. Spalletta and his colleagues studied 1015 AD patients, supporting 5 main syndromes based on the NPI (apathetic, affective, psychomotor, psychotic, and manic syndrome) that explained >76% of the variance (39). Reasons that the total explanation of the variance reached 76% may be as follows: first, the relatively large sample size, and the 1015 AD patients were newly diagnosed resulting in high homogeneity; second, the incidence of NPS in AD patients (especially for those with serious dementia) is higher than that in LLD patients, and the common symptoms were more concentrated in AD. In another study, Serra et al. recruited 101 AD patients and 56 MCI patients and extracted 5 main factors based on the NPI, accounting for 55.18% of the variance, which is similar to the present study (40). Therefore, not every study was able to explain a high percentage of the variance.

NPS factors may have different neurobiological underpinnings (37), and their effect on cognitive decline may be different. The most associated NPS factor with a mediating effect in patients with LLD has been underresearched, which limits early intervention possibilities. Our study supported the idea that behavioral factor, including disinhibition, agitation, irritability, and aberrant motor behavior symptoms, made the most critical contributions to the mediating effect on global cognition. Given that behavioral symptoms made great contributions to the mediation of global cognition, the mediation by information processing speed might be the combined effect of the three factors, indicating that emotional symptoms and psychotic symptoms were less associated with cognitive functions in this population with LLD. A recent publication using latent class analysis supported that irritability appeared to be the determining factor in the conversion to dementia in MCI samples (41). The neurobiological substrates underlying these relationships may be the association between irritability and lower fractional anisotropy in the anterior cingulate, which supports the idea that irritability was correlated with greater alterations in white matter hyperintensities. In a five-year follow-up study, more severe baseline agitation and aberrant motor behavior were associated with subsequent AD progression (42). The role of agitation in AD progression was clear in those with mild and moderate dementia (43), and aberrant motor behavior has been shown to predict mortality (44). We speculated that agitation, aberrant motor behavior, and progression of cognitive decline and functional deterioration in AD are related to dysfunction in the same brain regions, with some neurobiological changes connected with them (45). Disinhibition has rarely been evaluated, so little is known about its mediating effect on cognition, indicating the need for further investigations of disinhibition in the future. It is noteworthy that, to date, most of the related evidence on the neurobiology of the above behavioral symptoms, such as irritability and agitation, comes from the dementia literature with little data from those with LLD despite its prevalence. Assessments of biomarkers in CSF or other neuroimaging studies in LLD patients are needed to explore the mechanism by which behavioral symptoms exacerbate cognitive impairments in patients with LLD.

The present study also found a moderating effect of behavioral factor on the difference in MMSE scores between the aLLD and rLLD groups, suggesting that behavioral symptoms played a greater role in exacerbating cognitive impairments in rLLD. Therefore, cognitive impairment did not recover simultaneously with depressive symptoms during the recovery periods, which may be due to the continuous existence of NPS, especially the behavioral symptoms. Most of the studies addressing the presence of NPS have focused on emotional symptoms. For instance, O'Connor suggested that depression in older people is typically accompanied by lowered mood and great anxiety and agitation (46). The present findings showed that other NPS symptoms, especially behavioral symptoms, exerted a role, although anxiety and depression have been the most widely explored NPS in other studies. A possible explanation for our moderating effect is as follows: anxious and depressive symptoms included in emotional factor are likely to be reactive, temporary, and linked to the self-awareness of being cognitively and/or functionally affected. Thus, emotional symptoms may be more influential during acute episodes of LLD. When emotional symptoms tend to be stable during recovery periods, persistent behavioral symptoms start playing a more prominent role. What's more, we found that illness duration was not a moderating factor on the general cognition, as reported in previous studies (47, 48). We speculated that the reasons may be as follows: (1) the heterogeneity in the LLD group; (2) differential medication histories in rLLD and aLLD groups; and (3) subjective recall bias.

NPS are common in those with LLD, but few effective and safe treatments exist. The lack of reliable NPS measurement and lack of enough attention to the role of NPS in LLD may have mainly contributed to the situation. Our results suggested that the NPI might be an effective scale to assess NPS in those with LLD. Furthermore, our study is meaningful in optimizing the treatment strategies for LLD, emphasizing treating behavioral symptoms during the recovery periods. In addition to cognition enhancers, clinicians may indirectly improve cognitive functions in the long term by treating NPS as early as possible. Since there are currently no antidepressant drugs that can improve both emotional symptoms and cognitive symptoms (49), psychotherapy such as cognitive behavioral therapy (CBT) and some physical therapies such as transcranial magnetic stimulation (TMS) may be helpful. Antipsychotic drugs can also be applied if needed. We suggest that the whole-course management of depressive symptoms and other related core symptoms should be incorporated in the routine treatment for depression instead of focusing only on remission from depression.

There were some limitations in the present study. First, the study used scales to assess NPS and cognitive functions without incorporating biomarkers in CSF/blood samples or neuroimaging, such as positron emission tomography (PET), into the analysis. A few patients may have complicated the analysis with early neurodegeneration, resulting in mixed effects. Moreover, the mechanisms underlying the influence of NPS on cognition could not be clarified. Future studies incorporating these biomarkers will provide a deeper understanding of the underlying mechanisms. Second, the present study did not exclude the possible effect of drugs because many patients with LLD were taking variable doses of antidepressant medication. Third, due to the lack of dynamic assessment of patients' symptoms, the trajectory of symptom change was unclear. Thus, the dynamic relationship between depression episodes and cognitive functions still needs to be further explored. Fourth, as NPS in NC is not as common as in LLD patients, the unbalanced sample sizes between the groups might have influenced the statistical power. Last, the one-year follow-up may have been too short; thus, the predictive efficacy of NPS on cognitive decline needs to be further elucidated.

In summary, the present study showed that NPS (especially behavioral symptoms) exacerbated cognitive impairment in LLD patients and might contribute to residual cognitive impairments during recovery periods. Moreover, behavioral symptoms may serve as a potential predictive marker for LLD patients at higher risk for cognitive decline. Our findings provide a deeper understanding of the relationship between NPS and cognitive impairment in LLD patients and highlight that NPS should be comprehensively evaluated in clinical practice because of their significant effect on prognosis. Early evaluations and interventions for NPS, especially behavioral symptoms during recovery periods, are of great significance for improving the long-term prognosis of LLD patients.

## Data Availability Statement

The raw data supporting the conclusions of this article will be made available by the authors, without undue reservation.

## Ethics Statement

The studies involving human participants were reviewed and approved by the Ethics Committees of the Affiliated Brain Hospital of Guangzhou Medical University. The patients/participants provided their written informed consent to participate in this study.

## Author Contributions

MZ designed the study, analyzed and interpreted the data, and wrote the manuscript. BC and XZ designed the study, analyzed and interpreted the data, and revised the manuscript. YN designed the study and revised the manuscript. HZ, QW, NM, ZW, XC, QP, SZ, MY, and GL assessed the subjects. All authors contributed to the article and approved the submitted version.

## Funding

This study was supported by a grant from the National Natural Science Foundation of China (No. 81701341), the Guangzhou Municipal Psychiatric Diseases Clinical Transformation Laboratory (No: 201805010009), the Key Laboratory for Innovation Platform Plan, the Science and Technology Program of Guangzhou, China, the Science and Technology Plan Project of Guangdong Province (No. 2019B030316001), and the National Key Research and Development Program of China (No. 2016YFC0906300). The funders had no role in the study design, data collection and analysis, decision to publish, or preparation of the manuscript.

## Conflict of Interest

The authors declare that the research was conducted in the absence of any commercial or financial relationships that could be construed as a potential conflict of interest.

## Publisher's Note

All claims expressed in this article are solely those of the authors and do not necessarily represent those of their affiliated organizations, or those of the publisher, the editors and the reviewers. Any product that may be evaluated in this article, or claim that may be made by its manufacturer, is not guaranteed or endorsed by the publisher.
