# Supplementary material for: Neuropsychiatric Symptoms Exacerbate the Cognitive Impairments in Patients With Late-Life Depression
Source: Front Psychiatry. 2021 Nov 19;12:757003. doi: 10.3389/fpsyt.2021.757003 (PMC8639522; doi:10.3389/fpsyt.2021.757003)
Supplement: Supplementary file 1 [file Table_1.DOCX]

**Supplementary Table 1. Scores of NPI sub-factors between NC and LLD groups**

| variables | NC | LLD | t | *p* |
| --- | --- | --- | --- | --- |
| Emotional factor | -0.74±0.32 | 0.40±1.01 | -16.688 | p＜0.001 |
| Behavioral factor | -0.21±0.21 | 0.12±1.22 | -4.239 | p＜0.001 |
| Psychotic factor | -0.04±0.12 | 0.02±1.24 | -0.855 | 0.393 |

Notes: NC, normal controls; LLD, Late-life depression.

**Supplementary Table 2. Linear regression of correlations between NPS and cognitive functions**

|  | MMSE | Memory | Language ability | Information processing speed | Executive function | Visuospatial skill |
| --- | --- | --- | --- | --- | --- | --- |
| NPI-total |  |  |  |  |  |  |
| B | -0.39* | -0.15* | -0.15* | -0.14 | -0.08 | -0.15* |
| S.E. | 0.17 | 0.07 | 0.07 | 0.07 | 0.07 | 0.07 |
| 95% CI | -0.72 to -0.05 | -0.29 to -0.01 | -0.28 to -0.01 | -0.28 to 0.01 | -0.22 to 0.07 | -0.29 to 0.01 |
| β | -1.27 | -1.37 | -1.36 | -1.24 | -0.75 | -1.40 |
| Emotional factor |  |  |  |  |  |  |
| B | 3.98 | 1.71 | 1.63 | 1.35 | 0.65 | 1.78 |
| S.E. | 2.72 | 1.16 | 1.13 | 1.18 | 1.18 | 1.16 |
| 95% CI | -1.37 to 9.33 | -0.57 to 3.99 | -0.59 to 3.85 | -0.98 to 3.67 | -1.67 to 2.96 | -0.50 to 4.07 |
| β | 0.74 | 0.92 | 0.89 | 0.71 | 0.36 | 0.97 |
| Behavioral factor |  |  |  |  |  |  |
| B | 1.55 | 0.62 | 0.64 | 0.74 | 0.41 | 0.60 |
| S.E. | 1.13 | 0.47 | 0.46 | 0.48 | 0.48 | 0.47 |
| 95% CI | -0.67 to 3.76 | -0.31 to 1.56 | -0.26 to 1.55 | -0.20 to 1.69 | -0.53 to 1.35 | -0.33 to 1.54 |
| β | 0.27 | 0.29 | 0.31 | 0.34 | 0.21 | 0.28 |
| Psychotic factor |  |  |  |  |  |  |
| B | 0.53 | 0.41 | 0.45 | 0.24 | 0.14 | 0.34 |
| S.E. | 0.75 | 0.41 | 0.40 | 0.41 | 0.41 | 0.41 |
| 95% CI | -0.94 to 1.99 | -0.39 to 1.22 | -0.33 to 1.23 | -0.57 to 1.05 | -0.67 to 0.94 | -0.47 to 1.15 |
| β | 0.10 | 0.12 | 0.14 | 0.07 | 0.04 | 0.10 |
| Constant |  |  |  |  |  |  |
| B | 29.68*** | 2.26 | 2.15 | 2.05 | 1.19 | 2.26 |
| S.E. | 2.77 | 1.17 | 1.14 | 1.19 | 1.19 | 1.17 |
| 95% CI | 24.23 to 35.13 | -0.04 to 4.56 | -0.09 to 4.39 | -0.30 to 4.40 | -1.15 to 3.52 | -0.05 to 4.57 |
| Adjusted R^2^ | 0.23 | 0.16 | 0.17 | 0.19 | 0.11 | 0.14 |
| Model *p* | *** | *** | *** | *** | *** | *** |
| S.E. of the estimate | 4.69 | 1.59 | 1.56 | 1.60 | 1.56 | 1.60 |

Notes: NPI-total, total scores of Neuropsychiatric Inventory; MMSE, Mini-mental state examination; S.E., standard error; CI, confidence interval. * p <0.05; ** p <0.01; *** p <0.001.

**Supplementary Table 3. Demographic, clinical symptoms, neuropsychological and psychiatric symptoms**

| Variables | NC (n=141) | rLLD (n=192) | aLLD (n=70) | F/χ^2^/t | *p* value | *Post hoc* |
| --- | --- | --- | --- | --- | --- | --- |
| Demographic and clinical data | |  |  |  |  |  |
| Age | 67.72±5.42 | 68.69±7.14 | 67.71±6.74 | 1.118 | 0.328 | / |
| Sex (male/%) | 41(29.1%) | 44(22.9%) | 17(24.3%) | 1.680 | 0.432 | / |
| Education years | 10.76±3.03 | 8.33±4.10 | 7.51±3.95 | 24.166 | *p* <0.001 | A＞B, C |
| Age of onset | NA | 59.83±11.48 | 60.29±10.39 | -0.239 | 0.812 | / |
| Illness duration | NA | 5.87±8.79 | 3.95±4.07 | 1.401 | 0.163 | / |
| Numbers of episode | NA | 2.18±2.52 | 2.18±2.24 | 0.014 | 0.989 | / |
| HAMD | 1.81±2.37 | 8.23±4.75 | 22.97±5.80 | 562.602 | *p* <0.001 | C＞B＞A |
| Fluoxetine equivalents (unit) | NA | 0.54±0.60 | 0.50±0.56 | 0.482 | 0.630 | / |
| Cognitive functions |  |  |  |  |  |  |
| MMSE^#^ | 27.28±1.89 | 22.19±4.91 | 19.24±6.34 | 62.589 | *p* <0.001 | A＞B＞C |
| Memory^#^ | 1.04±1.19 | -0.76±1.65 | -1.26±1.48 | 59.413 | *p* <0.001 | A＞B, C |
| Language ability^#^ | 1.12±1.00 | -0.90±1.61 | -1.25±1.44 | 77.761 | *p* <0.001 | A＞B, C |
| Information processing speed^#^ | 1.01±1.29 | -0.72±1.73 | -1.17±1.48 | 49.758 | *p* <0.001 | A＞B, C |
| Executive function^#^ | 0.83±1.38 | -0.53±1.63 | -0.93±1.29 | 34.796 | *p* <0.001 | A＞B, C |
| Visuospatial skill^#^ | 0.97±0.62 | -0.70±1.78 | -1.16±2.26 | 42.336 | *p* <0.001 | A＞B, C |
| Neuropsychiatric symptoms | |  |  |  |  |  |
| NPI-total | 2.62±5.00 | 17.78±14.73 | 39.55±15.22 | 211.487 | *p* <0.001 | C＞B＞A |
| Emotional factor | -0.74±0.32 | 0.06±0.79 | 1.35±0.96 | 206.882 | P＜0.001 | C＞B＞A |
| Behavioral factor | -0.21±0.21 | 0.05±1.00 | 0.30±1.68 | 6.826 | 0.001 | C＞A, B |
| Psychotic factor | -0.04±0.12 | 0.00±1.00 | 0.10±1.74 | 0.467 | 0.627 | / |

Notes: NC, normal control; rLLD, recovery-state late life depression; aLLD, acute-state late life depression; HAMD, Hamilton Depression Rating Scale; NPI, Neuropsychiatric Inventory; MMSE, Mini-mental state examination; A, scores of NC group; B, scores of rLLD group; C, scores of aLLD group; ^#^ adjusted for age, sex, education years..
